# A randomized, placebo-controlled, cross-over trial of ketamine in Rett syndrome

**DOI:** 10.1186/s11689-025-09591-y

**Published:** 2025-01-24

**Authors:** Kathleen Campbell, Jeffrey L. Neul, David N. Lieberman, Elizabeth Berry-Kravis, Tim A. Benke, Cary Fu, Alan Percy, Bernhard Suter, David Morris, Randall L. Carpenter, Eric D. Marsh, Jana von Hehn

**Affiliations:** 1 Division of Developmental and Behavioral Pediatrics, Department of Pediatrics, Children’s Hospital of Philadelphia, Philadelphia, PA USA; 2 Department of Pediatrics, Vanderbilt Kennedy Center, Vanderbilt University Medical Center, Nashville, TN USA; 3 Department of Neurology, Boston Children’s Hospital, Boston, MA USA; 4 Rush University Medical Center, Chicago, IL USA; 5 Departments of Pediatrics, Pharmacology, Neurology and Otolaryngology, Children’s Hospital Colorado and University of Colorado School of Medicine, Aurora, CO USA; 6 School of Medicine, Department of Pediatrics, Neurology, Neurobiology, Genetics, and Psychology, University of Alabama at Birmingham, Birmingham, AL USA; 7 Texas Children’s Hospital, Baylor College of Medicine, Houston, TX USA; 8 WebbWrites, Durham, NC USA; 9 Rett Syndrome Research Trust, Trumbull, CT USA; 10 Division of Neurology, Department of Neurology, The Children’s Hospital of Philadelphia and University of Pennsylvania, Philadelphia, PA USA

**Keywords:** Rett syndrome, Ketamine, Clinical trial, Electroencephalography

## Abstract

**Background:**

Preclinical studies and anecdotal case reports support the potential therapeutic benefit of low-dose oral ketamine as a treatment of clinical symptoms in Rett syndrome (RTT); however, no controlled studies have been conducted in RTT to evaluate safety, tolerability and efficacy.

**Design:**

This was a sequentially initiated, dose-escalating cohort, placebo-controlled, double blind, randomized sequence, cross-over study of oral ketamine in 6–12-year-old girls with RTT to evaluate short-term safety and tolerability and explore efficacy.

**Methods:**

Participants were randomized to either five days treatment with oral ketamine or matched placebo, followed by a nine-day wash-out period and then crossed-over to the opposite treatment. Ketamine was dosed twice daily at 0.75 mg/kg/dose (Cohort 1) or 1.5 mg/kg/dose (Cohort 2). An independent safety monitoring committee evaluated safety and approved proceeding to the next dose cohort. Caregivers, participants, outcome assessors, and study staff except pharmacists were blinded to allocation. The primary endpoint was safety and tolerability. Exploratory efficacy endpoints included change in clinician- and caregiver-rated measures of RTT features, brain activity on electroencephalography, and wearable biosensors to measure respiration, heart rate, sleep, and activity.

**Results:**

Twenty-three participants enrolled (11 in Cohort 1, 12 in Cohort 2) from 3/12/2019–11/22/2021. One participant was excluded from analysis due to not meeting inclusion criteria on blinded review prior to analysis. One participant was withdrawn from the study due to an adverse event (vomiting) after the first dose of ketamine. Although planned for four dose cohorts, the trial was stopped after Cohort 2 due to enrollment challenges associated with the COVID-19 pandemic. Ketamine was safe and tolerated in both cohorts, with 1 related treatment emergent adverse event of vomiting. No difference was observed in efficacy between ketamine and placebo. Electroencephalography showed the expected increase in high frequency power with ketamine.

**Conclusions:**

Short-term, low-dose oral ketamine was safe and well tolerated in girls with RTT. No clinical efficacy of ketamine in treating symptoms of RTT was observed with 5 days of treatment, despite electroencephalography evidence of ketamine target engagement during the first dose. Further studies are needed to evaluate safety and efficacy of higher dose and longer exposure to ketamine in RTT.

**Trial registration:**

Registered at clinicaltrials.gov NCT03633058.

**Supplementary Information:**

The online version contains supplementary material available at 10.1186/s11689-025-09591-y.

## Introduction

Rett syndrome (RTT) is a rare, severe neurodevelopmental disorder that primarily affects females at an incidence of approximately 1 in 10,000 female births [1]. Variants in the gene methyl-CpG-binding domain 2 protein (*MECP2*) found on the X chromosome are causative in over 95% of cases [1]. RTT is characterized by apparently normal post-natal development for the first 6 months of life, followed by developmental delay, then regression of previously acquired skills. Main criteria for diagnosis include loss of purposeful hand use and speech, gait abnormalities, and stereotypic hand movements [1]. In addition, there are supportive criteria for diagnosing atypical RTT [1]. Beyond the main functional problems in RTT, seizures and constipation are also top caregiver concerns [2]. Animal models investigating neurotransmitter receptor dysfunction and excitatory/inhibitory balance have reproduced the developmental and autonomic features of RTT, allowing for investigation of potential therapeutics [3, 4].

In RTT mouse models, dysfunctional N-methyl-D-aspartate (NMDA) receptor activity is associated with disrupted excitatory/inhibitory balance of neural activity with brainstem hyperexcitability and forebrain hypoactivity [3, 4]. Ketamine, primarily an NMDA receptor antagonist [5], restores the excitatory/inhibitory balance, reverses hypoactivity in forebrain circuits, ameliorates regression and limb stereotypies, and extends life-span in mouse models with chronic daily administration (25 or 40 days), providing evidence ketamine may be therapeutic in RTT [6, 7]. Ketamine rapidly stimulates dendritic growth and translation and expression of key synaptic proteins regulated by brain-derived neurotrophic factor (BDNF) and mammalian target of rapamycin (mTOR) signaling [8], which have been shown to be deficient in *MECP2* mutants [9]. The effect of ketamine in animal models was sustained after conclusion of treatment, supporting hypotheses that ketamine can induce long-term changes in neuronal connectivity and brain function.

Limited studies of ketamine in humans with RTT have provided anecdotal evidence of efficacy. In a single case report presented at a conference, a 10-year-old RTT individual was given oral ketamine 0.75 mg/kg twice daily for 5 days for refractory seizures that resulted in seizure control as well as improvements in motor function, communication, and cognition, lasting 8 weeks before seizures re-emerged [10]. Repeated doses of ketamine conferred additional seizure control and sustained improvements in RTT features of motor function and communication. No randomized, blinded, placebo-controlled trials have been completed to test safety, tolerability, and efficacy in a larger group of girls with RTT.

Studies of ketamine in humans for other indications (depression, anxiety, pain) have demonstrated acceptable safety and tolerability [11]. Side effects reported from low dose, oral ketamine include dizziness, hallucinations, nausea, vomiting, drowsiness, confusion, lightheadedness, headache, somnolence, and anxiety [11, 12]. Due to extensive first-pass metabolism and approximately 20% oral bioavailability in humans, a starting oral dose of 0.75 mg/kg of ketamine is equivalent to an intravenous dose of approximately 0.15 mg/kg [11].The safety and efficacy of single oral doses up to 10 mg/kg [13] as well as repeated oral doses up to 1.5 mg/kg three times a day [12], have been demonstrated for pediatric indications.

Studying efficacy of a novel treatment in children with developmental disorders poses challenges in measuring effects. Clinician and caregiver rating scales provide important information about disorder severity. Additional measures of symptoms through algorithmic event detection and brain activity may provide objective quantitative measures. Biomarkers such as electroencephalography (EEG) and biosensors are less susceptible to bias and placebo effect but are still in an exploratory phase. Biomarkers can help determine a dose that engages the targeted brain activity before the likely time of clinical effect and guard against prematurely rejecting a potentially effective treatment. EEG has shown promise as a biomarker in studies of adults with anxiety and depression receiving a single dose of ketamine, reflecting immediate changes in power and long-term changes in more complex EEG measures which correlate to long-term clinical outcome [14]. In studies of typically developing adults with depression, ketamine induces brain changes detectable on EEG, including increased high frequency activity [15, 16], consistent with prior studies of the effect of low-dose ketamine on the brain in *MECP2*-null mice [6]. RTT has a known EEG signature of slower activity and a negative correlation between alpha/delta power ratio (a measure of relative middle frequency to low frequency power) and RTT severity [17, 18]. The known EEG differences in RTT provide potential targets for testing target engagement with ketamine and therapeutic effect.

Based on the motivating data in both animal models and humans to support the use of ketamine in RTT individuals, the current study aimed to evaluate the safety, tolerability and efficacy of sub-anesthetic low-dose oral ketamine compared with placebo in girls with RTT.

## Methods

### Study design and procedures

This was a double-blind, randomized, placebo-controlled, multi-center, sequential ascending dose cohort, cross- over study to evaluate the safety, tolerability, and efficacy of oral ketamine in girls with RTT and a confirmed pathogenic variant in methyl-CpG-binding protein 2 (*MECP2*), between 6–12 years of age (inclusive) who had not achieved menarche. The study planned for 4 cohorts initiated sequentially in ascending dose order (0.75 mg/kg BID, 1.5 mg/kg BID, 3 mg/kg BID, and 4.5 mg/kg BID for 5 days). Each cohort assessed one dose level of ketamine compared to placebo. At the conclusion of each cohort, an Independent Safety Monitoring Committee (ISMC) determined if there was adequate safety data to support initiation of the subsequent dosing cohort. Participants could participate in only one cohort.

**Fig. 1:**
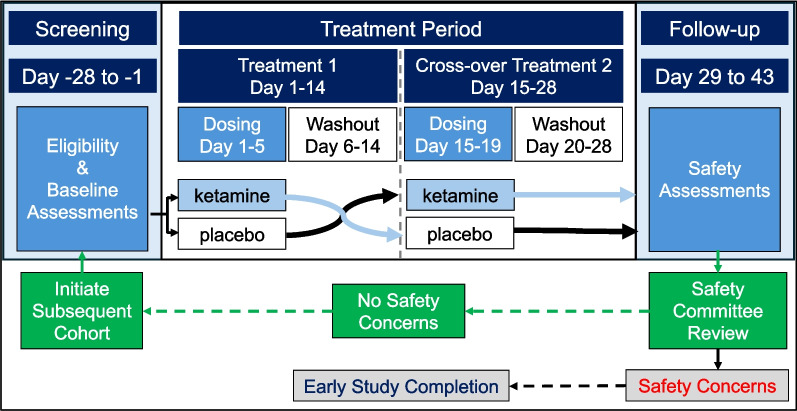
Study activities and safety monitoring

Study activities were divided (for each dose cohort) into 3 periods: Screening, Treatment, and Safety Follow-up. The Screening Period lasted between 14 and 28 days prior to the initiation of study drug. The Treatment period was a 4-week, double-blind, placebo-controlled, cross-over period to define safety and explore efficacy for the study. The Safety Follow-up Period was the final 2 weeks of the study to assess safety following Treatment Period completion (Fig. 1).

Study activities commenced only after the caregiver or legally authorized representatives provided appropriately obtained informed consent. Eligibility was confirmed at the Screening Visit, and participants received and trained on biosensors for daily in-home data collection prior to Day 1 Baseline Visit. At the Baseline Visit, participants continuing to meet eligibility criteria underwent in-clinic baseline safety and exploratory outcome evaluations (described below). Participants were centrally randomized through the electronic data capture system to treatment order (ketamine-placebo or placebo-ketamine) in a 1:1 ratio. Randomization was not stratified but was blocked by cohort. The randomization schedule was generated prior to study start.

The double-blind, placebo-controlled, cross-over Treatment Period lasted a total of 4 weeks, starting with the first treatment (ketamine or placebo) on Day 1 and initiation of the alternate treatment (cross-over) 2 weeks later on Day 15. Each ordered cross-over dosing regimen comprised 5 days of BID dosing, 9 days of washout, and participant safety and efficacy evaluation for 14 days after dose initiation (Period 1: Day 15, Period 2: Day 29). Participants received the first oral dose of each 5-day dosing regimen in-clinic under observation. If well tolerated, subsequent doses of the 5-day dosing regimen were administered in the home at approximately 12-h intervals, with or without food. The site contacted the caregiver each day of active dosing to confirm continued tolerability and to assess for emergent side effects.

After dosing completion and conclusion of the 4-week cross-over Treatment Period, participants returned on Day 29 for final safety and efficacy assessments. Efficacy assessments were completed by the same raters for each participant throughout the study. Participants were assessed for safety for an additional two weeks and a final Safety Follow-up phone call occurred on Day 43.

### Study population

This study (Ket-101-RSRT, NCT03633058), was conducted under IND 140628 at 7 sites in the United States (Children’s Hospital of Philadelphia, Vanderbilt University, Boston Children’s Hospital, Rush University Medical Center, Children’s Hospital of Colorado, University of Alabama at Birmingham, and Texas Children’s Hospital). Each institution received IRB approval prior to study initiation and no study procedures were conducted prior to obtaining informed consent.

Girls with typical or atypical RTT (according to the 2010 Clinical Diagnostic Criteria [1]) and a confirmed pathogenic variant in MECP2, between 6–12 years of age (inclusive) who had not achieved menarche were enrolled. Participants needed to be able to take liquid medications orally or through a feeding tube and had stable pharmacological (aside from anti-seizure medications) and non-pharmacological interventions for at least 4 weeks prior to Screening. Anti-seizure medical treatments were required to be stable for 12 weeks prior to Baseline Visit. Key exclusion criteria included uncontrolled epilepsy, planned changes to pharmacological or non-pharmacological interventions during the course of the study, history of prolonged QT syndrome or QTc prolongation on electrocardiogram, sensitivity to ketamine or concurrent treatment with medications that would interfere or interact with ketamine. Excluded medications included other NMDA antagonists, barbiturates, benzodiazepines, narcotics or opioids, sedatives other than melatonin or diphenhydramine, and strong CYP3A4 or CYP2B6 inducers and inhibitors.

### Dose selection

Four dose levels of ketamine were selected for assessment in this study: 0.75 mg/kg, 1.5 mg/kg, 3.0 mg/kg, and 4.5 mg/kg. Doses were selected to cover the sub-anesthetic range of ketamine, with the lowest dose having previously shown efficacy in an individual with RTT [10]. An ascending-dose cohort design was chosen to ensure the safety of each dose level cold be adequately determined before increasing the dose. Placebo solutions were dosed identically to ketamine-containing solutions.

### Treatment preparation and blinding

Investigational product (oral ketamine or placebo solution) was prepared by site investigational pharmacies according to compounding instructions in the Pharmacy Manual. Sterile commercial ketamine for intravenous/intramuscular injections (50 mg/ml or 100 mg/ml, West-Ward Pharmaceuticals/Hikma), was diluted with sterile water for injection to the specified volume, and a final solution consisting of 50% diluted ketamine and 50% masking agent (Ora-Sweet, Perrigo) was dispensed. Placebo consisted of 50% sterile water and 50% masking agent. Packaging, administration, and flavor masking between placebo and ketamine were identical.

Treatment order assignment was blinded to the study participants and all study personnel, aside from the unblinded study pharmacists for the duration of the study. At no time during the study was the blind broken. The ISMC received pre-specified unblinded datasets to assess safety prior to initiation of a subsequent cohort.

### Primary safety assessments

Safety was assessed through adverse events (AEs), serious adverse events (SAEs), concomitant medication use, vital signs (body temperature, heart rate, respiratory rate, oxygen saturation, and blood pressure), and physical exams. Participants were observed for 2 h after the initial in-clinic dose of each blinded treatment to assess for clinical concerns or side effects. Safety was subsequently evaluated in-clinic at all visits. Final safety assessment was made 2 weeks after completion of the study. Participants who experienced intolerable AEs, emergent adverse events, or other AEs considered to be related to study drug that reflected an unfavorable risk–benefit profile were withdrawn from the study at the discretion of the Investigator. Specific treatment-related AEs considered for withdrawal include Grade 3 (Severe) bradycardia, tachycardia, hypertension, hypotension, respiratory distress, syncope, changes in alertness, agitation, anxiety, mania, dizziness, anorexia, or nausea; or Grade 1 vomiting, hallucinations, or emergent delirium.

A medical monitoring committee consisting of the study investigators was convened on a regular basis to discuss AEs and overall interpretation of safety and tolerability based on blinded aggregate safety data. The ISMC reviewed unblinded safety data of a completed cohort to evaluate if the safety data supported initiation of the next ascending dose cohort.

### Exploratory efficacy assessments

Exploratory assessments of efficacy included clinician-rated and caregiver-rated measures, assessed at Baseline and the conclusion of each treatment period, and for some caregiver-rated assessments, mid-way through each treatment period and at the follow-up phone call (full schedule in Supplementary Table 1). Clinician-assessed measures included The Clinical Global Impression of Improvement (CGI-I), the Motor Behavior Assessment (MBA), and the Clinician Domain Likert Scale. The CGI-I is a 7-point Likert scale that captures the overall clinical impression of change from baseline after treatment, ranging from “Very Much Improved” (score = 1) to “Very Much Worse (score = 7) [19]. The RTT specific anchors for scoring the CGI-I were used [20]. The MBA is a 37-item scale that assesses a variety of clinical features in RTT and has been extensively captured in observational natural history studies of RTT [21]. Each item is scored between 0 (least severe or normal) and 4 (most severe), and a total MBA score calculated. The Clinician Domain Likert Scale was created for this study and involved evaluation of 8 RTT clinical domains (hand function, walking, verbal and non-verbal communication, comprehension, attention, behavior problems, mood), with each domain scored on a 7-point Likert scale to select one of seven numeric choices from “Normal (not at all impaired)” to “Extreme” for each domain. Investigators were trained on the use of scales prior to study start.

Caregiver-rated measures included the Rett Syndrome Behavior Questionnaire (RSBQ), Children’s Sleep Habits Questionnaire (CSHQ), the Parent Domain Likert Scale and Rett Syndrome Caregiver Burden Inventory Assessment (RTT CIA). The RSBQ is a 45-item measure to evaluate the behavioral and clinical features of RTT [22]. Ratings for each item are provided on a 3-point scale (0 = not true, 1 = somewhat or sometimes true, 2 = very true), and a total score is calculated by summing the individual items. The CSHQ was designed for children aged 4 through 12 years to screen for common sleep problems in that age group [23]. The RTT CIA, adapted from the Caregiver Burden Inventory created for Alzheimer disease to assess caregiver burden specifically for individuals with RTT [24], consists of 26 questions across 4 domains (time dependency, physical burden, emotional burden, social burden) appropriate for RTT and utilizes a 5-point Likert scale for answering each item: (1) I never feel this way, (2) I rarely feel this way, (3) I sometimes feel this way, (4) I quite frequently feel this way, (5) I nearly always feel this way. The Parent Domain Likert Scale was created for this study and is similar to the Clinician Domain Likert Scale with the inclusion of an additional domain, seizures.

### Exploratory biomarker assessments

EEG was collected on 10 participants at the time of the first in-clinic dose of each treatment at 3 of the sites where specific EEG equipment and expertise were available. Only sites that already had the necessary equipment and staff collected EEG to avoid excessive cost associated with new equipment and training. Participants watched a movie of their choice during the recording. After EEG electrode placement, participants were given at least 4 min to settle into a calm, resting state before the drug or placebo dose and EEG recording continued for 60 min after dose administration. The post-drug recording period was analyzed from 20–40 min after drug/placebo administration to capture the expected peak of drug concentration 30 min after drug administration [25]. EEG features before dose administration were used as a baseline to analyze change in EEG features after drug or placebo administration on the same day (to limit the impact of change in state or EEG lead placement on results).

### Biosensors

Participants wore two biosensors during the screening and treatment periods to capture activity, sleep, heart, and lung function. Specifically, the ActiGraph wGT3X-BT was used to capture activity, and the Hexoskin Smart Kit™ was used to capture activity, sleep, heart and lung function daily during the study.

### Statistical analysis

Sample size determinations were based on the primary outcome of safety and tolerability. For a dose limiting adverse event with a 10% incidence rate, a sample size of 10 participants/dose level (10 participants/cohort) provides a 65% probability of observing at least 1 event at each ketamine dose level, and an 88% probability of observing at least 1 event by completion of Cohort 2. For exploratory measures of efficacy, there are no reliable estimates of treatment effects of ketamine in RTT. Each dose level provided 80% power (α = 0.05, 2-sided paired t-test) to detect a 1.0 standard deviation treatment difference (large effect size) for exploratory efficacy assessments. Assuming a discontinuation rate ~ 15–20%, enrolling 12 participants in each cohort provides 10 participants completing the treatment period. All exploratory efficacy analyses were based on change from baseline.

The primary safety outcome analysis was performed on the Safety Population (*n* = 23), which included all participants who received at least one dose of study drug (ketamine or placebo). Exploratory efficacy analysis on clinical measures was performed on the Efficacy Population, which included all eligible participants who received both ketamine and placebo and had at least 1 post-treatment efficacy assessment (*n* = 21). No imputation was done for missing data, and no interim analysis was performed. A Statistical Analysis Plan for safety assessments and exploratory clinical efficacy assessments was finalized prior to database lock and breaking the blind.

All statistical analysis of safety data and exploratory clinical efficacy assessments was carried out in SAS®, Version 9.4. Study drug exposure was summarized by cohort and treatment for the duration of exposure (days), number of missed doses, and cumulative dose. Treatment-emergent adverse events (TEAEs) were defined as any adverse event (AE) that occurred after administration of the first dose of study drug. TEAEs were graded on severity (per Common Terminology Criteria for Adverse Events (CTCAE) v.4.03) and relationship to study drug. The number and percentage of participants who reported TEAEs were summarized by treatment at the time of the event by system organ class and preferred term, intensity, relationship, seriousness, and resulting discontinuation.

Statistical testing was performed for each ketamine dose cohort individually and combined. Analysis of treatment effect differences for exploratory clinical efficacy measures assessed at Baseline Visit (MBA, Clinician Domain Likert Scale, Parent Domain Likert Scale, RSBQ, CSHQ, RTT CIA) was performed on the change from Baseline (Day 1) to the end of the treatment period (Day 15 for first treatment and Day 29 for second treatment), and for caregiver-rated scales (Parent Domain Likert Scale, CSHQ, RTT CIA) mid-treatment (Day 8 for first treatment and Day 22 for second treatment) was also assessed. These were analyzed using an analysis of variance (ANOVA) model that included sequence (placebo-ketamine, ketamine-placebo), treatment, period, and treatment by sequence interaction as fixed effects and participant nested within sequence as a random effect. Sequence was tested using participant nested within sequence as the error term. Treatment by period was added into the model if period effect was found to be statistically significant; otherwise, it was not added in the model. Additionally, for CGI-I each participant was categorized as improved (at least minimal improvement per the CGI-I) or not improved and the McNemar test was used to assess treatment differences in the proportion of participants who improved on Day 15 and Day 29.

### EEG analysis

The two ketamine doses (0.75 mg/kg and 1.5 mg/kg) were combined to a single condition of drug administration for EEG analysis because only 10 participants had EEG collected. EEG was processed with MATLAB to remove periods of excessive movement, muscle activity, and poor signal as done previously [17]. There was no difference in amount of time rejected by processing in the drug condition compared with placebo, indicating that the EEG effect was not due to suppression of movement from sedating effects of ketamine (see supplementary methods). EEG analysis examined change in EEG frontal lobe power, resting state connectivity, alpha/delta ratio, interictal epileptiform discharges (IED), and EEG human rating after drug vs placebo administration. See supplementary methods for detailed description of EEG acquisition and analysis.

Statistical analysis of EEG effects was carried out in R version 2023.06.1 [26]. To check for bias in amount of time accepted in drug compared with placebo conditions, paired t-tests were used. Change in power, interictal epileptiform discharges (IED), correlation, and coherence after drug administration were analyzed with linear mixed effect models with drug as a fixed effect, channel nested in participant as a random intercept, and drug as a random slope to allow slopes to vary by group [27, 28]. The model with the random slope had a higher likelihood ratio than without it, and so was chosen as the best fit. Correlation between alpha/delta power ratio and clinical severity measures was carried out with Pearson correlation. Categorical rating of EEG by the human rater was compared in the drug vs placebo condition with chi-squared test. All statistical tests were two-sided and a *p*-value of 0.05 was considered statistically significant. No correction for multiple comparisons was applied.

## Results

### Participants

**Fig. 2:**
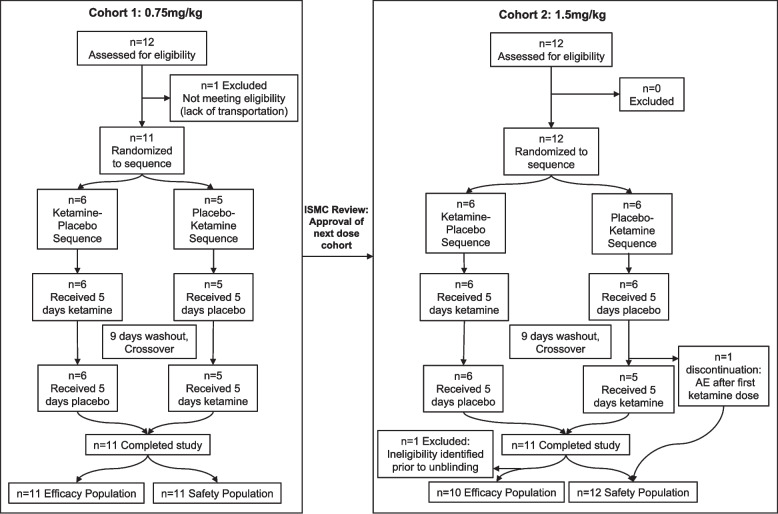
CONSORT diagram of study procedure and participant flow [29, 30]

The first participant enrolled on 12 March 2019 and the last participant completed the study on 22 November 2021. Although the trial was planned to include 4 sequential ketamine dose cohorts, enrollment challenges related to the COVID19 pandemic led to discontinuation of the trial after the second ketamine dose cohort (1.5 mg/kg BID for 5 days) was completed. Between the two dose cohorts, a total of 24 participants screened, 23 were enrolled, and one screen failed. Figure 2 presents the CONSORT diagram of the participant disposition for each dose cohort.

In Cohort 1 (0.75 mg/kg), 12 participants were screened, and one screen failed due to lack of reliable transportation to the site. Six participants were randomized to the ketamine-placebo sequence and five to the placebo-ketamine sequence. All 11 participants in Cohort 1 completed the study and were included in both the Safety Population and Efficacy Population analysis sets. Two participants missed one dose in the ketamine treatment period, and one participant received an extra dose on Day 6 (should have stopped on Day 5) in the ketamine treatment period. Six participants in Cohort 1 had EEG evaluations.

In Cohort 2 (1.5 mg/kg), 12 participants were screened and enrolled. Six participants were randomized to each treatment sequence group. One participant had treatment discontinuation within the placebo-ketamine sequence group due to experiencing a Grade 3 TEAE (vomiting) after receiving the first dose of ketamine in clinic but was included in the Safety Population analysis set. However, this participant was excluded from the Efficacy Population analysis set due to the pre-defined condition of lack of at least one efficacy assessment in both treatment periods. Additionally, a participant in the placebo-ketamine sequence group was found to be ineligible for study participation during blinded data review prior to database lock due to lack of stable anti-seizure medication in the 12 weeks prior to randomization and the presence of a second genetic disorder. There were no missed or extra doses during the ketamine treatment period in Cohort 2, but there was one missed dose in one participant during the placebo treatment period. Four participants in Cohort 2 had EEG evaluations.

In summary, the analysis for Cohort 1 included 11 participants in both the Safety and Efficacy Population analysis sets, Cohort 2 included 12 participants in the Safety Population set and 10 in the Efficacy Population, and for the overall study there were 23 participants in the Safety Population and 21 participants in the Efficacy Population. A total of 10 participants were included in the EEG analysis set.

### Baseline demographics and clinical characteristics

All but one participant (96%) met criteria for typical RTT and most (74%) carried 1 of the 8 most common *MECP2* point mutations. Table 1 provides the baseline demographics and clinical characteristics for participants included in the Safety Population set, split by cohort and treatment sequence assignment, as well as summarized for both cohorts by treatment sequence assignment. The majority of participants in all cohorts and treatment sequence groups were white (87%) and non-Hispanic (95.7%) and similar across all groups. Similarly, the age of participants was balanced between the cohorts and treatment sequence assignment groups.

**Table 1:** Participant characteristics

	**Cohort 1**		**Cohort 2**		**Both Cohorts**	
	**Ketamine-Placebo **(*n* = 6)	**Placebo-Ketamine **(*n* = 5)	**Ketamine-Placebo **(*n* = 6)	**Placebo-Ketamine **(*n* = 6)	**Ketamine-Placebo **(*n* = 12)	**Placebo-Ketamine **(*n* = 11)
**Demographics [Mean (SD)]**						
Age in years	9.3 (2.3)	7.8 (1.9)	7.5 (2.1)	7.7 (2)	8.4 (2.3)	7.7 (1.8)
Height in cm	130.7 (13.8)	120.3 (6.1)	116.6 (8.4)	118.2 (10.9)	124.3 (13.3)	119.1 (8.7)
Weight in kg	25.1 (7.7)	23.6 (7.7)	20.7 (3.8)	22.4 (5.6)	22.9 (6.2)	22.9 (6.3)
BMI in kg/m^2^	14.3 (1.4)	16.1 (4.3)	14.8 (1.8)	15.9 (1.9)	14.5 (1.5)	16 (3.1)
**Race [# (%)]**						
White	5 (83)	4 (80)	5 (83)	6 (100)	10 (83)	10 (91)
Black	0 (0)	1 (20)	0 (0)	0 (0)	0 (0)	1 (9)
Asian	0 (0)	0 (0)	1 (17)	0 (0)	1 (8)	0 (0)
Asian and white	1 (17)	0 (0)	0 (0)	0 (0)	1 (8)	0 (0)
Hispanic	0 (0)	0 (0)	0 (0)	1 (17)	0 (0)	1 (9)
Not Hispanic	6 (100)	5 (100)	6 (100)	5 (83)	12 (100)	10 (91)
**Baseline Severity [Mean (SD)]**						
CGI-S	4.8 (0.8)	4.4 (0.5)	4.7 (1)	4.7 (0.8)	4.8 (0.9)	4.5 (0.7)
MBA	47.5 (15.4)	44.4 (6.6)	48.7 (10.8)	45.3 (15.3)	48.1 (12.7)	44.9 (11.6)
ClinDom	35.2 (5.1)	34.4 (4.4)	32.8 (3.9)	31.5 (5.2)	34 (4.5)	32.8 (4.9)
RSBQ	37.8 (9.6)	39.8 (6.5)	44.3 (5.2)	47.7 (17.8)	41.1 (8.1)	44.1 (13.9)
CSHQ	81.5 (18.7)	91.2 (24.4)	107.5 (18)	108.3 (23.6)	94.5 (22.1)	100.5 (24.5)
ParDom	36.5 (6.6)	31.6 (6.4)	35.2 (10.1)	40 (7.9)	35.8 (8.2)	36.2 (8.2)
RTT CIA	19.5 (3.6)	40.4 (12.3)	40.3 (12.2)	39.5 (20.3)	29.9 (13.9)	39.9 (16.3)

*SD* Standard Deviation, *#* Number, *BMI* Body Mass Index, *CGI-S* Clinical Global Impression-Severity, *MBA* Motor Behavior Assessment, *ClinDom* Clinician Domain Likert Scale, *RSBQ* Rett Syndrome Behaviour Questionnaire, *CSHQ* Child Sleep Health Questionnaire, *ParDom* Parent Domain Likert Scale, *RTT CIA* Rett syndrome Caregiver Burden Inventory Assessment

Broadly, the baseline severity was matched between the treatment sequence assignment groups within cohorts and in both cohorts combined. The exception is the lower mean RTT CIA in the Ketamine-Placebo sequence group in cohort 1. Baseline diagnosed disorders by cohort are provided in Supplementary Table 2.

#### Primary endpoint analysis: safety and tolerability

##### All treatment emergent adverse events

Within both treatment cohorts, a total of 16 participants had at least one TEAE with any defined relationship to intervention (Table 2). In Cohort 1, two participants (2/11, 18.2%) had TEAEs (any relationship) during the ketamine treatment period, and four participants (4/11, 36.4%) had TEAEs (any relationship) during the placebo treatment period (Table 2). None of the participants in Cohort 1 had a TEAE that led to study discontinuation or dose interruption, or an SAE. In Cohort 2, seven participants (7/12, 58.3%) had TEAEs (any relationship) during the ketamine treatment period that led to treatment discontinuation in one participant, and three participants (3/12, 25.0%) had TEAEs during the placebo treatment period. Overall, a slightly higher percentage of participants experienced TEAEs during the ketamine phase compared to the placebo phase (39.1% versus 30.4%, respectively), though this was not considered to be clinically meaningful. No safety signals or TEAEs were observed in review of vital signs, including oxygen saturation levels. There were no deaths or SAEs in the study.

**Table 2:** All treatment emergent adverse events

**System Organ Class** Preferred Term	Cohort 1 (*n* = 11)		Cohort 2 (*n* = 12)		Both Cohorts (*n* = 23)	
	Placebo	Ketamine	Placebo	Ketamine	Placebo	Ketamine
	# (%)	# (%)	# (%)	# (%)	# (%)	# (%)
*Number of participants with at least one event*	4 (36.4)	2 (18.2)	3 (25.0)	7 (58.3)	7 (30.4)	9 (39.1)
**Gastrointestinal disorders**	2 (18.2)	1 (9.1)	1 (8.3)	3 (25.0)	3 (13.0)	4 (17.4)
Constipation	1 (9.1)	0	0	0	1 (4.3)	0
Diarrhea	0	0	0	1 (8.3)	0	1 (4.3)
Eructation	0	0	0	1 (8.3)	0	1 (4.3)
Vomiting	1 (9.1)	1 (9.1)	1 (8.3)	1 (8.3)	2 (8.7)	2 (8.7)
**General disorders and administration site conditions**	0	0	1 (8.3)	1 (8.3)	1 (4.3)	1 (4.3)
Pyrexia	0	0	1 (8.3)	1 (8.3)	1 (4.3)	1 (4.3)
**Infections and infestations**	0	0	0	2 (16.7)	0	2 (8.7)
Ear infection	0	0	0	1 (8.3)	0	1 (4.3)
Nasopharyngitis	0	0	0	1 (8.3)	0	1 (4.3)
**Injury, poisoning and procedural complications**	0	0	1 (8.3)	1 (8.3)	1 (4.3)	1 (4.3)
Stoma site irritation	0	0	1 (8.3)	0	1 (4.3)	0
Upper limb fracture	0	0	0	1 (8.3)	0	1 (4.3)
**Metabolism and nutrition disorders**	0	0	0	1 (8.3)	0	1 (4.3)
Decreased appetite	0	0	0	1 (8.3)	0	1 (4.3)
**Nervous system disorders**	1 (9.1)	0	2 (16.7)	2 (16.7)	3 (13)	2 (8.7)
Drooling	0	0	1 (8.3)	0	1 (4.3)	0
Sedation	0	0	0	1 (8.3)	0	1 (4.3)
Somnolence	0	0	1 (8.3)	1 (8.3)	1 (4.3)	1 (4.3)
Tonic convulsion	1 (9.1)	0	0	0	1 (4.3)	0
**Psychiatric disorders**	2 (9.1)	0	1 (8.3)	1 (8.3)	3 (13)	1 (4.3)
Insomnia	1 (9.1)	0	0	0	1 (4.3)	0
Irritability	1 (9.1)	0	0	1 (8.3)	1 (4.3)	1 (4.3)
Restlessness	0	0	1 (8.3)	0	1 (4.3)	0
**Respiratory, thoracic and mediastinal disorders**	0	1 (9.1)	1 (8.3)	0	1 (4.3)	1 (4.3)
Epistaxis	0	0	1 (8.3)	0	1 (4.3)	0
Rhinorrhea	0	1 (9.1)	0	0	0	1 (4.3)
**Skin and subcutaneous tissue disorders**	0	0	0	2 (16.7)	0	2 (8.7)
Nail discoloration	0	0	0	1 (8.3)	0	1 (4.3)
Skin irritation	0	0	0	1 (8.3)	0	1 (4.3)

The most common TEAE (any relationship) System Organ Class (SOC) across both treatment cohorts was gastrointestinal disorders experienced by 4 participants (4/23, 17.4%) during the ketamine treatment compared to 3 participants (3/23, 13%) during placebo treatment (Table 2). Other TEAE (any relationship) SOC groups were experienced by only 1–2 participants for both cohorts or within each individual cohort and were similar in frequency between the treatment periods. Overall, the majority of TEAEs (any relationship) were Grade 1 (mild) or Grade 2 (moderate) in severity (25/27, 92.6%), with the exception of the Grade 3 severity TEAE (vomiting) after receiving the first dose of ketamine that led to treatment discontinuation.

##### Treatment emergent adverse events related to intervention

The majority of related TEAEs observed were Grade 1 (mild) or Grade 2 (moderate) in severity (12/13, 92.3%), with the exception of the Grade 3 severity TEAE (vomiting) after receiving the first dose of ketamine that led to treatment discontinuation. This event occurred in a participant in Cohort 2 who was allocated to the placebo-ketamine treatment sequence. After receiving the first dose of ketamine (1.5 mg/kg) in clinic, the participant vomited approximately 15 min later and was subsequently withdrawn by the investigator (blinded to treatment at the time of the event). There were no other participants in either cohort that had a related TEAE that led to dose interruption.

The most frequent related TEAEs observed were in Nervous System Disorders and Psychiatric Disorders experienced by 2/23 participants (8.7%) during ketamine treatment and 2/23 participants (8.7%) during placebo treatment. In the overall study (both cohorts), the frequency of related TEAE SOCs was very similar during the placebo treatment period and the ketamine treatment period. Psychiatric disorder related TEAEs were more frequent in the placebo treatment period in Cohort 1. In Cohort 2, during the ketamine treatment period, one participant had related TEAE of an upper limb fracture (1/12, 8.3%), one had decreased appetite (1/12, 8.3%), and one had nail discoloration (1/12, 8.3%). Interestingly, the frequency of Nervous System Disorders and Psychiatric Disorders was similar in Cohort 2 between the placebo treatment period and the ketamine treatment period, despite the potential neuropsychiatric effects that might occur with ketamine treatment.

In summary, ketamine treatment appeared to be safe and well tolerated at the two doses tested (0.75 mg/kg or 1.5 mg/kg BID) in participants with RTT, with most TEAEs being Grade 1–2 severity and relatively similar in frequency during placebo and ketamine treatment, except one Grade 3 vomiting event after the first dose of ketamine leading to treatment discontinuation.

#### Exploratory efficacy endpoints

The exploratory clinical efficacy endpoints did not show any change from baseline with ketamine treatment compared to placebo treatment in any of the defined measures for the combined cohorts (Table 3), or for either of the cohorts when analyzed individually. The ANOVA model also included the assessment of a Sequence or Treatment-Sequence interaction. No Sequence effect was observed for any of the measures, and no Sequence-Treatment interaction was observed except for the Parent Domain Likert Scale, which was significant for the combined cohorts (*p* = 0.008) and Cohort 2 (*p* = 0.0284), but not for Cohort 1 (*p* = 0.194). This interaction was driven by a decreased score (improvement) during ketamine treatment in the placebo-ketamine sequence group versus a decreased score during placebo treatment in the ketamine-placebo sequence group. Neither change was significantly different from the baseline scores nor viewed as clinically meaningful.

**Table 3:** Clinical efficacy of ketamine compared with placebo for both cohorts combined

Both Cohorts (*n* = 21)				
Measure	**Placebo** **LSM (SE)**	**Ketamine** **LSM (SE)**	**Difference** **(95% CI)**	**Treatment** **(*p*-value)**
**MBA**	44.56 (2.63)	45.57 (2.63)	1.01 (−0.73, 2.75)	0.2373
**CGI-I**	3.81 (0.09)	3.79 (0.09)	−0.01 (−0.22, 0.19)	0.8895
**ClinDom**	33.25 (0.96)	33.67 (0.96)	0.42 (−0.63, 1.46)	0.4149
**RSBQ**	38.04 (2.77)	38.97 (2.77)	0.93 (−2.13, 3.99)	0.5315
**CSHQ**	106.39 (5.90)	99.19 (5.90)	−7.19 (−16.48, 2.09)	0.1213
**ParDom**	33.58 (1.93)	34.29 (1.93)	0.71 (−0.97, 2.39)	0.3877
**RTT CIA**	34.50 (3.44)	33.54 (3.44)	−0.96 (−3.73, 1.81)	0.4779

*MBA* Motor Behavior Assessment. Higher score indicates more severe RTT symptoms. *CGI-I* Clinical Global Impression-Improvement. Lower scores indicate more improvement. *ClinDom* Clinician Domain Likert Scale. Higher scores indicates more severe Rett symptoms. *RSBQ* Rett Syndrome Behavior Questionnaire. Higher score indicates more severe RTT symptoms. *CSHQ* Children’s Sleep Habits Questionnaire. Higher score indicates more sleep problems. *ParDom* Parent Domain Likert Scale. Higher scores indicates more severe Rett symptoms. *RTT CIA* Rett Caregiver Burden Inventory Assessment. Higher score indicates higher caregiver burden. *LSM* Least Square Mean, *SE* Standard Error, *CI* Confidence Interval

The CGI-I was also evaluated based on the number (percentage) who had at least minimal improvement (CGI-I score of 3 or less) after placebo or ketamine treatment. In Cohort 1, two participants showed minimal improvement after placebo treatment (2/11, 18.2%) compared to one participant after ketamine treatment (1/11, 9.1%, *p* = 0.021, placebo greater than ketamine). In Cohort 2, two participants showed minimal improvement after placebo treatment (2/10, 20%) compared to three participants after ketamine treatment (3/10, 30%, *p* = 0.096). Overall, there did not seem to be any benefit of ketamine treatment in the exploratory measures assessed. Analysis of biosensor data for sleep, heart rate variability, and breathing similarly revealed no difference between ketamine and placebo (data not shown).

#### Exploratory neurophysiological biomarker endpoints

##### EEG effects of ketamine

**Fig. 3:**
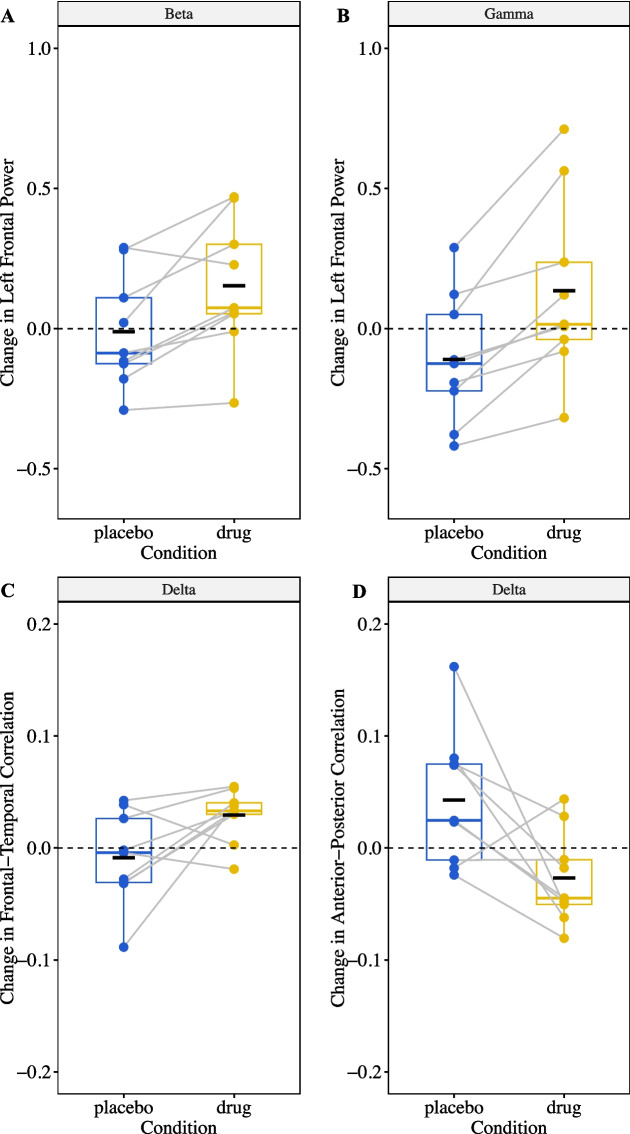
Change in EEG measures after administration of drug compared with placebo. Black solid horizontal lines represent means, black dashed line represents zero change, gray solid lines connect paired samples from the same participants

Although 5 days of ketamine dosing did not change clinical symptoms of RTT compared with placebo, we expected ketamine to have an immediate effect of increased high frequency brain activity [15, 16]. To further investigate whether the doses were sufficient to change brain activity we examined the EEG for signs of target engagement in the brain. After ketamine administration, high frequency (beta and gamma) activity in the left frontal lobe increased (Table 4; Fig. 3A, B). Additionally, left frontal 1/f slope became more positive (Table 4), consistent with the shift in the power spectrum toward greater high frequency power. In right frontal lobe no change in power was observed with ketamine compared with placebo.

**Table 4:** Change in EEG power and connectivity after administration of drug vs placebo

Measure	Fixed Effect: Drug vs Placebo		
Frequency Band	**Coefficient**	**(95% CI)**	***P*-value**
**Left Frontal Power**			
Delta	−0.005	(−0.11, 0.10)	*p* = 0.92
Theta	−0.02	(−0.09, 0.05)	*p* = 0.59
Alpha	0.02	(−0.05, 0.08)	*p* = 0.62
Beta	0.14	(0.06, 0.22)	***p* = 0.001**
Gamma	0.24	(0.14, 0.33)	***p* < 0.001**
1/f	0.006	(0.002, 0.01)	***p* = 0.004**
**Right Frontal Power**			
Delta	−0.04	(−0.27, 0.06)	*p* = 0.55
Theta	−0.10	(−0.10, 0.06)	*p* = 0.23
Alpha	−0.04	(−0.18, 0.10)	*p* = 0.58
Beta	0.07	(−0.09, 0.23)	*p* = 0.40
Gamma	0.13	(−0.05, 0.31)	*p* = 0.23
1/f	0.004	(−0.001, 0.009)	*p* = 0.09
**Frontal–Temporal connectivity**			
Delta	0.04	(0.001, 0.08)	***p* = 0.04**
Theta	0.02	(−0.03, 0.07)	*p* = 0.32
Alpha	0.01	(−0.03, 0.06)	*p* = 0.57
Beta	−0.01	(−0.03, 0.003)	*p* = 0.10
Gamma	−0.01	(−0.03, 0.01)	*p* = 0.28
**Anterior–Posterior connectivity**			
Delta	−0.07	(−0.13, −0.01)	***p* = 0.03**
Theta	−0.06	(−0.12, 0.001)	*p* = 0.05
Alpha	0.02	(−0.01, 0.05)	*p* = 0.15
Beta	−0.005	(−0.02, 0.01)	*p* = 0.49
Gamma	−0.004	(−0.02, 0.01)	*p* = 0.64
**Left–Right connectivity**			
Delta	−0.01	(−0.05, 0.03)	*p* = 0.50
Theta	−0.07	(−0.02, 0.03)	*p* = 0.34
Alpha	0.02	(−0.02, 0.05)	*p* = 0.24
Beta	−0.01	(−0.05, 0.03)	*p* = 0.50
Gamma	−0.02	(−0.05, 0.02)	*p* = 0.28

In exploratory analysis to test for other effects of ketamine on the brain in RTT, some measures of functional connectivity changed with drug compared with placebo. Frontal–temporal correlation increased and anterior–posterior correlation decreased after administration of ketamine compared with placebo in the delta frequency band only (Table 4; Fig. 3B, C). Mean delta frequency frontal–temporal correlation was 0.26 (SD 0.04) before drug and increased to 0.29 (SD 0.04) after drug. Mean delta frequency anterior–posterior correlation was 0.22 (SD 0.06) before drug and decreased to 0.20 (SD 0.03) after drug. Left–Right correlation did not change significantly with drug administration compared with placebo. Frontal–temporal, anterior–posterior, and left–right coherence did not change with drug administration compared with placebo.

##### EEG correlation with clinical measures

**Fig. 4:**
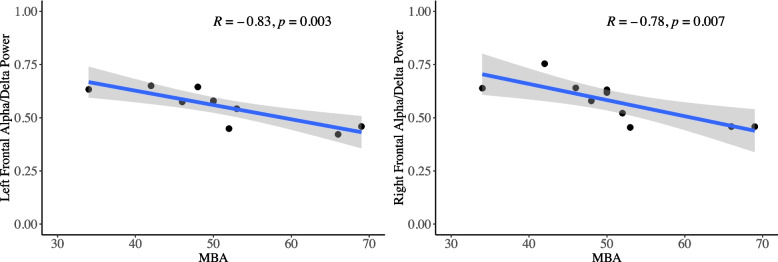
Correlation between alpha/delta power ratio and MBA at baseline. Overlayed text shows Pearson correlation and *p*-value. Blue line represents linear model, shaded area represents 95% confidence interval of linear model

EEG analysis of the alpha/delta power ratio demonstrated the expected relationship with RTT severity but did not change with drug. The alpha/delta power ratio at first EEG pre-drug/placebo was significantly negatively correlated with clinical severity (MBA) in the left and right frontal lobes (Fig. 4; left Pearson correlation −0.83, 95% CI −0.96, −0.42, *p* = 0.003; right Pearson correlation −0.78, 95% CI, −0.95, −0.30, *p* = 0.007). The alpha/delta ratio did not change after drug compared with placebo administration (coefficient 0.008, 95% CI −0.024, 0.040, *p* = 0.61).

### Interictal Epileptiform Discharges (IED) and human rating of EEG abnormalities

Change in IED/minute was not significantly different in drug compared with placebo in any channel. No differences were noted in the drug compared with placebo condition in any rating category by the human rater (Supplementary Table 3). 80% of EEGs had disorganized background in drug and placebo, 20% had increased interictal discharges after drug and 10% after placebo, 10% had improved state after drug and placebo, sleep was noted in 30% after drug and 40% after placebo, and no seizures were recorded.

## Discussion

In this randomized, placebo-controlled crossover trial, oral ketamine was well-tolerated in 6–12-year-old females with RTT at two low sub-anesthetic doses. Ketamine produced a detectable, immediate change in brain activity demonstrating target engagement. However, the effect of ketamine on RTT features and severity after 5 days of exposure to ketamine was no different from placebo. Possible reasons for the lack of efficacy could be that the doses tested, though high enough to demonstrate target engagement on EEG, were below the threshold for inducing measurable therapeutic effects or the study was not long enough for measurable change in symptoms. The two planned higher sub-anesthetic ascending dose cohorts (3.0 mg/kg and 4.5 mg/kg) were not tested due to enrollment challenges. Future studies could address these shortcomings with higher doses and longer treatment durations to determine whether ketamine has a therapeutic effect in RTT.

### Safety

Oral, low-dose ketamine had few mild side effects at the doses studied here except for vomiting in 1 participant at the time of treatment initiation that was pre-defined for participant withdrawal. Including the withdrawn participant, three participants (3/23, 13%) experienced 4 vomiting events. Two events on placebo and one on ketamine were deemed unrelated to treatment and were grade 1. Two prior pediatric studies of oral low dose ketamine in other disorders noted vomiting in one participant who received 1.5 mg/kg oral ketamine and in one participant who received 5 mg/kg oral ketamine [12, 31]. Vomiting, swallowing dysfunction, gastroesophageal reflux, and other gastrointestinal disorders are common in RTT and were present in 87% of participants at baseline (Supplementary Table 2) [32]. Psychiatric effects were notably not present more often than placebo (although reporting of symptoms was indirectly through the caregiver due to limited communication in RTT). No other adverse events were observed that may limit use of ketamine for treating features of RTT in future studies.

### EEG biomarkers of ketamine effect

The effect of ketamine observed in participants with RTT in this study, increased high frequency power, is consistent with prior studies of low-dose ketamine in *Mecp2*-null mice and typically-developing adults with anxiety and depression [6, 16, 33]. 1/f slope increased significantly, and non-significantly lower coefficients were observed in the models of low frequency power. Increased 1/f slope with ketamine indicates a shift toward more high frequency power and less low frequency power. 1/f slope is known to be more negative in RTT compared with typically developing controls and in individuals with RTT who have already regressed compared to those who have not yet regressed or are currently regressing [17, 18]. 1/f slope also correlates with developmental scales, with a more positive 1/f slope correlating to a higher developmental quotient [18]. Studies of boys with Fragile X syndrome found that higher resting gamma power in frontal lobes was correlated with better language skills [34]. Further investigation is needed to determine whether the increased gamma power and 1/f slope would be sustained with continued ketamine dosing and eventually result in a change in developmental outcomes. EEG biomarkers of drug effects in the brain may be useful to guide future dose-finding and understanding of the expected clinical impact of a drug.

The alpha/delta power ratio, a measure of relative middle frequency to low frequency power, was correlated with RTT severity on the MBA. This finding replicates a known correlation in RTT, suggesting that our clinical sample and EEG measures are consistent with others [17]. Alpha/delta power ratio did not change with ketamine compared with placebo. Therefore, future studies may need longer duration of ketamine dosing to determine the impact on clinical features and the relationship to alpha/delta power ratio.

Low frequency functional connectivity (correlation) decreased in anterior–posterior areas and increased in frontal–temporal areas with ketamine administration compared with placebo. Diffuse low frequency (delta) synchronization is a known atypical pattern in neurodevelopmental disorders, [35] therefore, reversal of this pattern may be therapeutic but this hypothesis would need further testing. In a study of cannabidiol, reduced anterior–posterior connectivity was associated with drug response in children with intractable epilepsy treated with cannabidiol [36]. Reductions in hyper-connectivity in frontal-posterior cingulate networks has also been suggested as a potential mechanism for antidepressant effect of ketamine in functional MRI studies of the default mode network [37]. Increased functional connectivity in frontal–temporal areas may be related to normalization of resting state activity, as it has been similarly detected on functional MRI associated with normalization of atypical language function in autism spectrum disorder [38]. Future work with a typically developing control group or longitudinal analysis is needed to understand whether the observed changes in functional connectivity with ketamine is countering atypical brain development in RTT.

Change in EEG activity was apparent by quantitative EEG measures despite no apparent change detected by an expert human rater. Quantitative EEG measures are a useful biomarker of engagement of brain activity with drug that is not apparent to human experts and may precede clinical effect.

### Limitations

There are several potential limitations to consider in interpreting the results of this study including the ability to only assess the 2 lowest planned doses, and the short 5-day duration of treatment. Additionally, enrollment challenges due to the COVID-19 pandemic limited the sample size so that testing of the 2 planned higher doses was not possible and may have impacted the ability to recruit a more diverse study participant population which may limit generalizability.

As the EEG analysis was exploratory, we tolerated a potential beta error and included effects on the EEG that may be related to chance. Some of the EEG data was excluded by the artifact rejection algorithm due to participant movement and muscle activity, leaving an average of 2 min pre-drug/placebo and 9 min post-drug/placebo for analysis, though approximately 1 h was collected on each participant. A minimum amount of EEG data for accurate quantitative EEG measures has not been fully established, although the amount of EEG data kept in this study was similar to other studies of children with RTT and CDKL5 deficiency disorder, and replicates known clinical correlations [18, 39]. Given the small sample and 2 similar doses we did not compare EEG effects between doses. Future studies with a wider dose range and larger sample could include dose comparisons.

### Conclusion and future directions

In this study of girls with RTT between the ages of 6 and 12 years, low dose oral ketamine was well tolerated without a significant change in clinical features. The clinical effect of ketamine was not significantly different from baseline or placebo treatment. This study established an immediate effect of ketamine on EEG in humans with RTT, but it is not yet known whether long-term dosing would have a sustained effect at this dose or if a higher dose would be needed. Future studies could use a longer treatment duration to test whether a ketamine drug effect is associated with an important long-term clinical effect.

## Supplementary Information

Supplementary Material 1.

## Data Availability

The data that support the findings of this study are available on reasonable request from the corresponding authors [JLN, JvH, EDM]. The data are not publicly available due to containing information that could compromise research participant privacy.

## Abbreviations

ANOVA: Analysis of Variance
AE: Adverse Event
BID: Twice a day
BMI: Body Mass Index
CGI-I: Clinical Global Impression-Improvement
CGI-S: Clinical Global Impression-Severity
CI: Confidence Interval
CSHQ: Children’s Sleep Habits Questionnaire
CTCAE: Common Criteria for Adverse Events
CYP: Cytochrome P450
EEG: Electroencephalogram
IED: Interictal Epileptiform Discharges
ISMC: Independent Safety Monitoring Committee
LSM: Least Square Mean
MBA: Motor Behavior Assessment
MECP2: Methyl-CpG-binding protein 2
NMDA: N-methyl-D-aspartate
RSBQ: Rett Syndrome Behavior Questionnaire
RTT: Rett Syndrome
RTT CIA: Rett Syndrome Caregiver Burden Inventory Assessment
SD: Standard Deviation
SE: Standard Error
TEAE: Treatment Emergent Adverse Event
