# Supplementary material for: A randomized, placebo-controlled, cross-over trial of ketamine in Rett syndrome
Source: J Neurodev Disord. 2025 Jan 24;17:4. doi: 10.1186/s11689-025-09591-y (PMC11761732; doi:10.1186/s11689-025-09591-y)
Supplement: Supplementary file 1 — Supplementary Material 1. [file 11689_2025_9591_MOESM1_ESM.docx]

**Supplementary Materials**

Supplementary Table 1: Schedule of Clinical Questionnaires

| **Study Period** | **Pre-treatment** |  | | **Treatment Period 2** | | | **Safety** |
| --- | --- | --- | --- | --- | --- | --- | --- |
|  |  | **Treatment Period 1** | | |  | |  |
|  | Screening | Visit 2 |  | Visit 3 |  | Visit 4 | Follow-up |
| **Measure** | Day -28 to -1 | Day 1 | Day 8 | Day 15 | Day 22 | Day 29 | Day 43 |
| **MBA** |  | X |  | X |  | X |  |
| **CGI-S** | X |  |  |  |  |  |  |
| **CGI-I** |  | X |  | X |  | X |  |
| **ClinDom** |  | X |  | X |  | X |  |
| **RSBQ** |  | X |  | X |  | X |  |
| **CSHQ** |  | X | X | X | X | X |  |
| **ParDom** |  | X | X | X | X | X | X |
| **RTT CIA** |  | X |  | X |  | X | X |

CGI-S: Clinical Global Impression-Severity; CGI-I: Clinical Global Impression-Improvement; MBA: Motor Behavior Assessment; ClinDom: Clinician Domain Likert Scale; RSBQ: Rett Syndrome Behaviour Questionnaire; CSHQ: Child Sleep Health Questionnaire; ParDom: Parent Domain Likert Scale; RTT CIA: Rett syndrome Caregiver Burden Inventory Assessment.

**Supplementary EEG methods:**

*EEG Acquisition*

EEG equipment consisted of 21 gold-plated electrodes placed using the international 10-20 system. EEG data were recorded with Natus Neuroworks EEG system software (Natus Medical Incorporated, Middleton, WI). Data were recorded at a sampling frequency of 256 Hz at 2 sites and 512 Hz at 1 site. The drug or placebo was administered 4-66 minutes after initiation of EEG recording, EEG recording lasted 60-156 minutes.

*Data Processing*

EEG files were exported as European Data Format and imported into Matlab (v2022a, Mathworks, Natick, MA) for processing and analysis. Custom scripts were used to perform spatial and frequency filtering, artifact rejection, and extract quantitative EEG measures including power, correlation, and coherence as previously performed (3,4). Briefly, raw- machine referenced EEG for each patient was spatially filtered to a Laplacian average reference and frequency filtered to 1-70 Hz with a second-order Butterworth bandpass filter and a notch filter from 59.5 to 60.5 Hz. Non-overlapping segments of 0.5 second were automatically included or rejected for artifacts from movement, muscle activity, or line noise. Automated rejection algorithms included absolute signal amplitude greater than 150 microvolts, root mean square amplitude more than 2 standard deviations from the mean root mean square amplitude of each 0.5 second segment, or line length greater than 2.5 standard deviations from the median absolute deviation line length of each 0.5 second segment. For power calculations, rejection was performed per channel to allow artifact-free data to be kept in a channel even if an artifact was detected in another channel so that local effects of muscle did not influence rejection of data in distant electrodes. For correlation and coherence calculations, all channels were rejected if an artifact was detected in one channel to avoid biasing measures that require comparison of coincident channel activity. Artifact-free usable segments were divided into 4-second epochs for analysis.

*Power*

For each 4-second epoch, mean power spectral density was calculated using a Fast Fourier transform with a rectangular window (Matlab function periodogram). The power spectral density was then integrated using the trapz function in frequency bins, specifically delta (1-4 Hz), theta (4-8 Hz), alpha (8-13 Hz), beta (13-25 Hz), and gamma (26-59 Hz). Power calculations were log-transformed and averaged within each frequency bin. Slope of the power spectral density, 1/f, was calculated by fitting a linear slope to the curve. Change in median power was calculated for drug and placebo in each frequency bin and in 1/f for frontal electrodes (left F3, F7, C3, Fp1; right F4, F8, C3, Fp2;). The ratio of median alpha power to delta power was computed for comparison of the ratio of high to low frequency power to the clinical severity measure, the Motor Behavior Assessment (MBA).

*Correlation and Coherence*

Resting state connectivity between brain regions was quantified by calculating correlation for power-based connectivity and coherence for phase-based connectivity. Correlation and coherence give a measurement between 0 and 1, with 0 signifying no synchrony and 1 signifying perfect synchrony (5). Correlation was calculated with Spearman correlation coefficients (corr function) to obtain correlation of amplitude between regions within each frequency band followed by the mean of the absolute value of the diagonal of the correlation matrix. Coherence was calculated with the MATLAB mscohere function, which calculates magnitude-squared coherence using Welch’s overlapped averaged periodogram. The mean coherence was calculated for each 4-second segment within each frequency band. Change in correlation and coherence after drug and placebo were quantified between regions of interest including frontal-temporal (electrodes Fp1, F3, Fp2, F4 to T3, T5, T4, T6), left-right (Fp1, F3, C3, P3, F7, T3, T5, O1 to Fp2, F4, C4, P4, F8, T4, T6, O2), and anterior-posterior (Fp1, Fp2 to O1, O2 and F3, F4 to O1, O2).

*Analysis of Time Accepted vs Rejected by EEG processing*

In the analysis of EEG power, there was no difference between time accepted by the channel-level artifact rejection algorithm in drug compared with placebo conditions (p=0.55 pre, p=0.37 post), indicating that results were not biased by an effect of the drug reducing movements (sedating) in patients receiving ketamine. Average EEG time analyzed before placebo/drug administration was 1.69 minutes (SD 1.34, range 0.12-3.81) in the placebo condition and 2.09 minutes (SD 1.16, range 0.27-3.87) in the drug condition. Average time after placebo/drug administration was 9.91 minutes (SD 6.76, range 2.12-24.43) in the placebo condition and 7.75 minutes (SD 5.43, range 1.04-15.94) in the drug condition. One subject was excluded for insufficient data (less than 28 seconds) after drug administration, based on current standards for minimum acceptable time to estimate power (1,2).

In the analysis of EEG functional connectivity, there was no difference between time accepted in drug compared with placebo conditions (p=0.83 pre, p=0.58 post), indicating the results were not biased by an effect of the drug reducing movements (sedating) in patients receiving ketamine. EEG time accepted before placebo/drug administration for resting state connectivity was 0.81 minutes (SD 0.7, range 0.07-2.07) in the placebo condition and 0.89 minutes (SD .79, range 0.13-2.8) in the drug condition. The average time accepted after placebo/drug administration was 3.55 minutes (SD 2.96, range 0.13-7.93) in the placebo condition and 3.07 minutes (SD 2.96, range 0.2-9.87) in the drug condition.

*Interictal Epileptiform Discharges*

Interictal epileptiform discharge (IEDs, spikes and sharp waves) were defined as discharges with a pointed peak and amplitude distinct from the background that last less than 200 milliseconds and are separated from each other by at least 150 milliseconds (6). IEDs were detected as outliers from the signal by smoothing with a moving mean and smoothing factor of 0.25, then subtracting the peaks from the smoothed signal, taking the absolute value of the peaks, and passing the peaks through a hampel filter set to detect the outliers more than 5 standard deviations from the local root mean square amplitude. Automatic IED detection was compared with detection by a human electrophysiologist (EDM) on a sample of 10 randomly selected 8-second segments of EEG from 10 patients in the placebo condition. Sensitivity of automatic IED detection was quantified by true positives/(true positives + false negatives) and accuracy was quantified by true positives/(true positives + false negatives + false positives). Sensitivity of automatic IED detection was moderate compared with human rating (77%, 95% CI 57%, 89%) and accuracy was low (19%, 95% CI 13%, 28%).

*Human reading of EEG*

An experienced neurophysiologist (CA) reviewed the EEGs and rated features including background, sharp waves, change in state, sleep, and seizures. The rater was blinded to drug or placebo condition but was informed of the time of drug or placebo administration.

*Analysis of target engagement*

Non-rejected epochs of EEG data before drug/placebo administration (pre) and after drug/placebo administration (post) were quantified. Up to 5 minutes from the pre-drug segment were captured starting at least 4 minutes after the start of recording to allow time for the subject to reach a comfortable steady state. The post-drug recording period was marked from 20 minutes to 40 minutes after drug/placebo administration to capture the expected peak of drug concentration, 30 minutes after drug administration (7). Change in power, correlation, coherence, and IEDs was calculated by subtracting the mean (or median) pre-drug from post-drug value for each subject.

Supplementary Table 2: Baseline medical conditions.

| **System Organ Class** Preferred Term, [# (%)] | **Cohort 1 (n=11)** | **Cohort 2 (n=12)** | **Both Cohorts (n=23)** |
| --- | --- | --- | --- |
| **Eye disorders** | 4 (36.4) | 0 | 4 (17.4) |
| Astigmatism | 2 (18.2) | 0 | 2 (8.7) |
| Strabismus | 2 (18.2) | 0 | 2 (8.7) |
| **Gastrointestinal disorders** | 10 (90.9) | 10 (83.3) | 20 (87.0) |
| Constipation | 10 (90.9) | 7 (58.3) | 17 (73.9) |
| Gastrooesophageal reflux disease | 4 (36.4) | 3 (25.0) | 7 (30.4) |
| **Immune system disorders** | 5 (45.5) | 3 (25.0) | 8 (34.8) |
| Food allergy | 2 (18.2) | 1 (8.3) | 3 (13.0) |
| Seasonal allergy | 3 (27.3) | 2 (16.7) | 5 (21.7) |
| **Musculoskeletal and connective tissue disorders** | 5 (45.5) | 2 (16.7) | 7 (30.4) |
| Hip deformity | 2 (18.2) | 0 | 2 (8.7) |
| Scoliosis | 5 (45.5) | 2 (16.7) | 7 (30.4) |
| **Nervous system disorders** | 5 (45.5) | 7 (58.3) | 12 (52.2) |
| Epilepsy | 1 (9.1) | 5 (41.7) | 6 (26.1) |
| Hypotonia | 1 (9.1) | 1 (8.3) | 2 (8.7) |
| Seizure | 2 (18.2) | 2 (16.7) | 4 (17.4) |
| **Psychiatric disorders** | 4 (36.4) | 7 (58.3) | 11 (47.8) |
| Anxiety | 4 (36.4) | 3 (25.0) | 7 (30.4) |
| Insomnia | 1 (9.1) | 1 (8.3) | 2 (8.7) |
| Sleep disorder | 0 | 2 (16.7) | 2 (8.7) |
| **Skin and subcutaneous tissue disorders** | 2 (18.2) | 1 (8.3) | 3 (13.0) |
| Eczema | 2 (18.2) | 1 (8.3) | 3 (13.0) |
| **Surgical and medical procedures** | 7 (63.6) | 5 (41.7) | 12 (52.2) |
| Gastrostomy | 4 (36.4) | 3 (25.0) | 7 (30.4) |
| Hip surgery | 2 (18.2) | 0 | 2 (8.7) |

Supplementary Table 3: Human expert rating of EEG in drug compared with placebo.

|  | **Drug**  (n=10) | **Placebo**  (n=10) | **p-value** |
| --- | --- | --- | --- |
| **Background Organization**  Disorganized  Normal  Underdeveloped | 8 (80%)  1 (10%)  1 (10%) | 8 (80%)  0 (0%)  2 (20%) | 0.51 |
| **Sharp Waves**  Abundant  Frequent  Occasional  Rare  None | 2 (20%)  2 (20%)  2 (20%)  1 (10%)  3 (30%) | 0 (0%)  3 (30%)  3 (30%)  0 (0%)  4 (40%) | 0.47 |
| **Increased interictal discharges**  Present  Absent | 2 (20%)  8 (80%) | 1 (10%)  9 (90%) | 1 |
| **Change in State**  Improved  No change  Uncertain  Worse | 1 (10%)  7 (70%)  1 (10%)  1 (10%) | 1 (10%)  8 (80%)  0 (0%)  1 (10%) | 0.79 |
| **Sleep**  Present  Absent  Not clear | 3 (30%)  6 (60%)  1 (10%) | 4 (40%)  5 (50%)  1 (10%) | 0.89 |
| **Seizures**  Present | 0 (0%) | 0 (0%) | 1 |

**References:**

1. Salinsky MC, Oken BS, Morehead L. Test-retest reliability in EEG frequency analysis. Electroencephalography and Clinical Neurophysiology. 1991 Nov 1;79(5):382–92.

2. Sathyanarayana A, El Atrache R, Jackson M, Cantley S, Reece L, Ufongene C, et al. Measuring Real-Time Medication Effects From Electroencephalography. J Clin Neurophysiol. 2022 May 17;

3. Armstrong C, Zavez A, Mulcahey PJ, Sogawa Y, Gotoff JM, Hagopian S, et al. Quantitative electroencephalographic analysis as a potential biomarker of response to treatment with cannabidiol. Epilepsy Research. 2022 Sep 1;185:106996.

4. Saby JN, Benke TA, Peters SU, Standridge SM, Matsuzaki J, Cutri-French C, et al. Multisite Study of Evoked Potentials in Rett Syndrome. Annals of Neurology. 2021;89(4):790–802.

5. Cohen MX. Analyzing Neural Time Series Data: Theory and Practice [Internet]. 1st ed. Cambridge, MA: The MIT Press; 2014. Available from: https://doi.org/10.7551/mitpress/9609.001.0001

6. Kane N, Acharya J, Benickzy S, Caboclo L, Finnigan S, Kaplan PW, et al. A revised glossary of terms most commonly used by clinical electroencephalographers and updated proposal for the report format of the EEG findings. Revision 2017. Clin Neurophysiol Pract. 2017;2:170–85.

7. Grant IS, Nimmo WS, Clements JA. Pharmacokinetics and Analgesic Effects of I.M. and Oral Ketamine. British Journal of Anaesthesia. 1981 Aug;53(8):805–10.
